# Unveiling New Molecular Factors Useful for Detection of Pelvic Inflammatory Disease due to *Chlamydia trachomatis* Infection

**DOI:** 10.5402/2012/581725

**Published:** 2012-10-14

**Authors:** Carmen Rodriguez-Cerdeira, Elena Sanchez-Blanco, Alberto Molares-Vila, Alfonso Alba

**Affiliations:** ^1^Dermatology Department, CHUVI and University of Vigo, 36210 Vigo, Spain; ^2^Predoctoral Researcher in Health Sciences, University of Vigo, 36210 Vigo, Spain; ^3^Analytical Chemistry Department, University of Vigo, 36210 Vigo, Spain; ^4^Department of Molecular Biology, Centre for Molecular and Cellular Studies, Lugo, Spain

## Abstract

*Background*. Untreated *Chlamydia trachomatis* infections in women can result in disease sequelae such as pelvic inflammatory disease (PID), ultimately culminating in tubal occlusion and infertility. While nucleic acid amplification tests can effectively diagnose uncomplicated lower genital tract infections, they are not suitable for diagnosing upper genital tract pathological sequelae. *Objective*. The purpose of this paper was to provide a comprehensive review of new molecular factors associated with the diagnosis and prognosis of PID. *Material and Methods*. The literature was searched using the key words “*Chlamydia trachomatis* infections,” “pelvic inflammatory disease,” and “molecular factors” in the PubMed database. Relevant articles published between 1996 and 2012 were evaluated. *Conclusions*. The use of new molecular factors could potentially facilitate earlier diagnosis and prognosis in women with PID due to *C. trachomatis* infection.

## 1. Introduction

Pelvic inflammatory disease (PID) is a polymicrobial infection of the upper genital tract (UGT). It primarily affects young, sexually active women. The diagnosis is made clinically; no single test or study is sensitive or specific enough for a definitive diagnosis. PID should be suspected in at-risk patients who present with pelvic or lower abdominal pain with no identified etiology and who have cervical motion, uterine, or adnexal tenderness. *Chlamydia trachomatis *is one of the commonly implicated bacterial microorganisms; however, other microorganisms may be involved. The spectrum of disease ranges from asymptomatic to life-threatening tuboovarian abscess. Patients should be treated empirically, even if they present with few symptoms. Most women can be treated successfully as outpatients with a single dose of a parenteral cephalosporin plus oral doxycycline, with or without oral metronidazole [1]. Delay in treatment may lead to major sequelae, including chronic pelvic pain, ectopic pregnancy, and infertility. Hospitalization and parenteral treatment are recommended if the patient is a pregnant woman [1, 2].

The microorganisms that are implicated in PID are thought to spread in the following three ways:

- intra-abdominally, traveling from the cervix to the endometrium, through the salpinx, and into the peritoneal cavity (causing endometritis, salpingitis, tuboovarian abscess, or pelvic peritonitis);
- through the lymphatic systems, for example, infection of the parametrium from an intrauterine device (IUD);
- through hematogenous routes, for example, with tuberculosis, although this is rare.

The diagnosis of PID is based primarily on clinical evaluation. Because of the potential for significant consequences if treatment is delayed, physicians should treat patients on the basis of clinical judgment without waiting for confirmation from laboratory or imaging tests [3].

The objective of this study is to analyze molecular factors that may help to make the diagnosis and prognosis of PID in the different stages of the disease.

## 2. Materials and Methods

A systematic review was conducted using PubMed of the National Center for Biotechnology Information (NCBI).

The article search focused on covering all scientific publications of PID and related molecular factors published between 1996 and 2010. Reference lists of PID publications were utilized to identify relevant literature and reviewed for completeness of already found publications. No attempt was made to identify unpublished studies. Ethical approval was not sought, since the study relied on published data only.

The study selection was done in two stages: during the first phase, all publications involving a component of PID and molecular factors were included. Publications focusing on the molecular biology of PID were also selected. The study selection at this point was done using abstracts or full publications if the abstract did not give sufficient information. At the second phase, complete publications were reviewed and their suitability with respect to the research objective was assessed. Case-series and conference abstracts were excluded at the second stage of review.

## 3. Results and Discussion

### 3.1. *Chlamydia* Pathogenesis

The cellular paradigm of *Chlamydia* pathogenesis [4] states that the host response to chlamydiae is initiated and sustained by epithelial cells, which are the primary targets of chlamydial infections. Infected host epithelial cells act as first responders, initiating and propagating immune responses [5]. They secrete chemokines that recruit inflammatory leukocytes to the site of infection and cytokines that induce and augment the cellular inflammatory response [6], and these mediators induce direct damage to the tissues. At the time of reinfection, host cell release of chemokines leads to recruitment of *Chlamydia*-specific immune cells that rapidly amplify the response. The release of proteases, clotting factors, and tissue growth factors from infected host cells and infiltrating inflammatory cells leads to tissue damage and eventual scarring—the hallmark of *Chlamydia*-induced oviduct disease. The cellular paradigm makes no distinction between damage induced by professional innate immune cells (neutrophils and monocytes) and adaptive lymphocyte populations but assumes that both cell populations contribute to the pathogenesis. Chronic chlamydial infections are common [7] and would lead to ongoing release of mediators that promote continued influx of inflammatory cells, damage to host epithelium, scarring, and, ultimately, fibrosis and scarring. Because reinfection with chlamydiae occurs frequently [8], repeated inflammatory responses may lead to repeated insults to the tissues and may promote tissue scarring.

### 3.2. Molecular Factors

Among the molecular factors reviewed is the *Chlamydia* heat shock protein 60 (cHSP60), which has been investigated as a potential antigen responsible for the induction of delayed type hypersensitivity-induced disease. Later studies conducted in a guinea pig model of trachoma revealed a protective role for vaccination with cHSP60 [9]. Although human studies have revealed elevated antibody counts to cHSP60 in those with more severe disease [10, 11], this may simply reflect increased exposure to *Chlamydia* through chronic or repeated infection. A recent large prospective study of women with PID did not reveal a correlation of increased antibody counts to cHSP60 with worse outcome [12]. 

In a prospective cohort study involving women at high risk of *C. trachomatis* infection, Cohen et al. [13] found that at baseline and after adjustment for age and other potential confounding variables, production of interferon (IFN)-*γ* by peripheral-blood mononuclear cells (PBMCs) stimulated with cHSP60 strongly correlated with protection against incident *C. trachomatis* infection.

Debattista et al. [14] found that low PBMC IFN-*γ* and high interleukin (IL)-10 responses to cHSP60 were markers for increased risk of chlamydial infection and PID. In human immunodeficiency virus-seropositive women, a CD4 lymphocyte count of <400 cells/mm^3^ was determined to be an independent risk factor for *C. trachomatis* PID [15]. *Chlamydia*-specific CD4 T1 helper cell (Th1)-IFN-*γ*-producing cells are key mediators of host defense; a goal for vaccine development should be to determine *Chlamydia* antigens and adjuvants that induce a strong CD4 Th1 memory response [5]. A persistent cHSP60 antibody response was correlated with having culture- or ligase chain reaction-positive oviduct samples after treatment, which suggests that antibody positivity is a useful marker of chronic infection [16]. These data indicate that prolonged or repeated exposure to chlamydiae leads to increased risk for disease and increased detection of anti-chlamydial antibodies, rather than directly implicating antibody formation in the pathogenesis. Although high antibody responses to cHSP60 have been correlated with increased susceptibility to chlamydial PID [10, 15], IFN-*γ* responses to this highly conserved protein have been correlated with protection among the same group of women [15].

Researchers have begun to determine the cellular receptors involved in *C. trachomatis*-induced stimulation of cytokine release. Toll-like receptors (TLRs) act as pathogen-recognition receptors that enable cells to recognize conserved bacterial, viral, and fungal structural elements. In vitro, *C. trachomatis* infection of HEK cells transfected with the adaptor molecule MyD88 and the pathogen molecular pattern receptors TLR2 and TLR4/MD-2 revealed that TLR2 was required for IL-8 secretion and that the role of TLR4/MD-2 was minimal. This was reproduced with chlamydial infection of immortalized human ectocervical epithelial cells [17]. The response was largely dependent on the MyD88 adaptor molecule. Confocal microscopy experiments revealed that both TLR2 and MyD88 colocalize with the intracellular chlamydial inclusion, suggesting that TLR2 is actively engaged in signaling from this intracellular location. There is a protective role for TLR2 deficiency in genital tract infection sequelae due to *C. trachomatis* [5]. Examination of human tissue samples for the various TLRs has revealed that the mRNA for TLR2 is highly expressed in Fallopian tubes and the cervix [18]. Thus, TLR2 may be a primary pathogen-recognition receptor available in the lower genital tract and oviducts to drive the pathology-inducing inflammatory response to chlamydial infection [5].

Whilst nucleic acid amplification tests can effectively diagnose uncomplicated lower genital tract (LGT) infections, they are not suitable for diagnosing UGT pathological sequelae. Several studies have demonstrated a correlation between antibody responses to cHSP60 and pathologic sequelae in women [19–21], including a significant association between the presence of antibodies to cHSP60 and PID [10, 21, 22]. These data have led to the development of a commercial enzyme-linked immunosorbent assay (ELISA) screening test based on cHSP60 (Medac, Hamburg, Germany). Studies evaluating the diagnostic potential of the Medac cHSP60 ELISA test have demonstrated conflicting results, and thus the ability of the cHSP60-based assay to distinguish various *C. trachomatis* disease stages may be limited [23, 24].

Witkin et al. have identified several chlamydial antigens that could be used to discriminate between uncomplicated LGT infection and UGT pathology due to *C. trachomatis* [25]. Four amino acid bands allow physicians to distinguish between LGT infection and UGT pathology in affected women. Two possible candidates were identified for each of band A (CT147 and CT314), B (CT727 and CT396), and C (CT157 and CT423). Band A, reactive in 38% of *C. trachomatis*-infected samples, was identified as two possible candidate proteins: CT147 (conserved hypothetical protein: 162.1 kDa) and CT314 (DNA-directed RNA polymerase beta chain: 154.9 kDa). Only CT147 has previously been shown to elicit a humoral response as expected from the protein's localization to the inclusion membrane of the elementary body (EB) [26]. CT314 functions as a transcriptional regulator and would not be expected to be presented to the host immune system at any stage during the chlamydial developmental cycle or infection process. 

The two candidate proteins for band B are CT727 (P-type ATPase) and CT396 (HSP70). P-type ATPases constitute a superfamily of cation transport enzymes that mediate transmembrane exchange of all biologically significant cations [27]. In contrast, HSP70 is associated with outer membrane complexes of EBs and was originally thought to play a role in either attachment or entry of the EB into host cells [28, 29]. It is suggested that HSP70 indeed confers a humoral antibody response.

One of the candidate proteins for band C, CT157, contains two phospholipase D (PLD) domains and is a member of the PLD superfamily, which includes enzymes that have high catalytic activity and are involved in phospholipid metabolism. PLDs, which are known to hydrolyze phospholipids to phosphatidic acid, may be essential for the formation of particular types of transport vesicles or be strongly involved in signal transduction [30]. CT423, the second protein candidate for band C, contains three functional domains (two CBS domains and one transporter-associated domain) that are implicated in intracellular targeting and trafficking as well as protein-protein interactions [31]. 

Sensitivity and specificity of the identified antigens in various combinations showed the A or B or C format to be the most efficacious for diagnosing uncomplicated LGT infection. The addition of antigen D to the panel (A or B or C or D) was shown to increase the sensitivity to 79%. However, given the overall prevalence of antigen D in samples from *C. trachomatis*-infected patients, the diagnostic potential of antigen D for specifically identifying LGT infections is limited due to the high *C. pneumoniae* cross-reactivity demonstrated within UGT patients. Moreover, this suggests that antigen D could possibly be more useful as a marker of general chlamydial infections rather than of a particular stage of infection [25]. 

A small study conducted by Kuo et al. [32] showed that the chemokine receptor deletion mutation CCR5-Δ32 correlated significantly with protection from tubal damage. CCR5 is crucial for T-cell activation and function. 

Endocervical epithelial cells released IL-1*α* after infection, and the induced proinflammatory cytokine cascade could be inhibited by specific anti-IL-1*α* antibodies [6]. The addition of an IL-1 receptor antagonist to the cultures completely eliminated tissue destruction induced by infection, indicating a direct role for this cytokine in the pathogenesis [33].

Other potentially important factors are matrix metalloproteinases (MMPs), which are expressed by neutrophils and monocytes and are involved in proteolysis and resynthesis of extracellular matrix. Studies in humans also indicate a role for MMPs and neutrophils in the pathogenesis of tissue damage. Fallopian tube epithelial cells infected in vitro with *C. trachomatis* produce MMP-2, and infected oviduct stromal cells produce MMP-9 [34].

An interrelated protease mechanism involves two interesting markers, cathepsin B and cystatin C. Cathepsin B belongs to the family of lysosomal cysteine proteases and is active in acidic environments [35]. Cathepsin B is a lysosomal protease and located inside lysosomes. It has also been found to be secreted as an extracellular contributor to degrade extracellular matrix (ECM) molecules [36] or as a regulator involved in cell death modulation [37, 38]. It has been shown that cathepsin B mediates hepatic inflammation and injury caused both by apoptosis and the production of proinflammatory chemokines [39]. Previous studies have shown that cathepsin B plays a critical role in the tumor necrosis factor (TNF)-*α*-triggered apoptotic cascade and promotes cell death through participation in the extrinsic pathway in which caspase-8 causes the release of active cathepsin B from lysosomes; consequently, cathepsin B increases the cytosol-induced release of cytochrome C from mitochondria [40, 41]. In contrast, Nagai and his colleagues found that cathepsin B could inhibit neuronal cell death that was induced by cystatin C [42]. However, Foghsgaard et al. found that proteolytic enzyme families, for example, cathepsin B and cysteine proteases, regulate apoptosis and play opposite roles in malignancies by reducing tumor cells by means of their proapoptotic features and by enhancing tumor cells through their known facilitation of invasion [38].

Cystatin C, an endogenous cysteine protease inhibitor, is a nonglycosylated low molecular weight (13 kDa) secretory protein produced by nucleated cells. It has been found in a variety of human tissues but is mainly found in extracellular body fluid and serum [43–45]. Cystatin C is associated with the regulation of inflammation [46] and cell death [42]. Clinically, a patient's altered cystatin C level in bodily fluid or serum is monitored or used to predict the progression of diseases [47–50]. A high concentration of cystatin C has been reported in patients with hepatic disease, and it has therefore been suggested that cystatin C could be used as a marker for monitoring liver functions and the progression of liver fibrosis [50]. Cystatin C is also used as a predictor for the reduction in kidney function, which may be associated with increased inflammation or adverse pathophysiological consequences [51, 52]. Tsai et al. [53] have found a significantly increased expression of cathepsin B but a decreased expression of cystatin C as well as an imbalance in the equilibrium between cathepsin B and cystatin C in patients with PID. Thus, significantly low levels of cystatin C and significantly high levels of cathepsin B in the serum of patients with PID before they received treatment were found. In addition, the ratio of the cathepsin B level to the cystatin C level in the serum of patients with PID increased significantly before the patients received treatment compared with after they had received treatment according to the protocol suggested by the Centers for Disease Control and when compared with healthy controls. Although this regulatory mechanism needs further investigation, it has been suggested that the detection of serum levels of cathepsin B and cystatin C, as well as the serum ratio of cathepsin B to cystatin C, can provide useful clinical information for PID.

From the bacterial point of view, nine surface-exposed *C. trachomatis* polymorphic membrane proteins (Pmps) are encoded via a multigene family yielding PmpA to PmpI [54]. Pmps represent 13.6% of the coding capacity of the *C. trachomatis* genome [54], suggesting that they have a critical role in biology and virulence [55, 56]. These findings imply either a role for these specific Pmps in inflammation or simply that women with PID have sustained and increased exposure due to repeated or chronic infection [55]. Taylor et al. [57] have suggested that PmpA plays a role in the pathology of UGT, although these data were nonsignificant. In addition, PmpD may stimulate host cell inflammatory responses, and it is possible that an increased antibody titer to PmpD reflects increased exposure to these potentially pathogenic ligands. In the study by Taylor et al. [57], increased inflammation and reproductive sequelae were found among women with high antibody titers to PmpD. However, these results were also nonsignificant. Overall, expression of the PmpD antibody appeared to have minimal effects on inflammation and reproductive sequelae in this study. In addition, the authors found that women with antibody reactivity to PmpI were more likely to have UGT infection (UGTI) [57]. Endometritis was also more frequent in this group, although these results were nonsignificant (Table 1).

## 4. Conclusions

Several molecular factors have been investigated in the past years for their application in the early detection and identification of chronic PID caused by *C. trachomatis* infection.

Although there is already a diagnostic method (Medac cHSP60 ELISA test), its utility is limited, and there is no other commercial method known to date. 

The other discussed host molecular factors are thought to be of interest as new potential markers in the diagnosis at different stages of the disease; however, further investigation and clinical trials will have to be carried out.

Bacterial molecular factors comprise another focus of interest. Membrane proteins in *C. trachomatis*, which are known to be related to inflammation and chronic PID, may be candidates for commercial antibody development for avoiding harmful infections.

## Figures and Tables

**Table 1:** Different molecular factors for detection of pelvic inflammatory diseases (PID).

Name	Symbol	GenBank ID (http://www.ncbi.nlm.nih.gov/gene/)
Heat shock protein 60	cHSP60	3329

Toll-like receptor 2	TLR2	7097

CT147 hypothetical protein	CT147	884191

rpoC DNA-directed RNA polymerase subunit beta	CT314	884808

zntA metal transport P-type ATPase	CT727	884518

dnaK molecular chaperone DnaK	CT396	884718

CT157 phospholipase D endonuclease	CT157	884104

CT423 CBS domain-containing protein	CT423	884688

Chemokine (C-C motif) receptor 5 (gene/pseudogene)	CCR5-Δ32	1234

Interleukin 1, alpha	IL-1*α*	397094

Matrix metallopeptidase 2 (gelatinase A, 72 kDa gelatinase, 72 kDa type IV collagenase)	MMP-2	4313

Matrix metallopeptidase 9 (gelatinase B, 92 kDa gelatinase, 92 kDa type IV collagenase)	MMP-9	4318

Cathepsin B	CTSB	1508

Cystatin C	CST3	1471

Polymorphic outer membrane protein	pmpA	884704; 7883162; 5858107; 7881799;
		5859597; 12593919; 12387900;
		12387048; 11729968; 3687904;
		13015005; 13016481; 13013794

Polymorphic outer membrane protein	pmpI	7882069; 884675; 5859284; 7882474;
		5858752; 3687560; 12594420;
		12387544; 12388396; 11730441;
		13015593; 13014300; 13016052

Polymorphic outer membrane protein	pmpD	884611; 5858550; 7881652; 7882759;
		5859752; 12594356; 12387479;
		12388331; 11730376; 3687805;
		13015983; 13014231; 13015529
